# Observer perceptions of the justifiability of the actions of nations in conflict: The relative importance of conveying national vulnerability versus strength

**DOI:** 10.1371/journal.pone.0220303

**Published:** 2019-07-29

**Authors:** Kimberly Matheson, Nyla Branscombe, Yechiel Klar, Hymie Anisman

**Affiliations:** 1 Department of Neuroscience, Carleton University, Ottawa, Ontario, Canada; 2 Institute of Mental Health Research, University of Ottawa, Ottawa, Ontario, Canada; 3 Department of Psychology, University of Kansas, Lawrence, Kansas, United States of America; 4 Department of Psychology, University of Tel Aviv, Tel Aviv, Israel; Valparaiso University, UNITED STATES

## Abstract

Because the underdog in a conflict typically gains the support of observers, nations will often adopt a narrative that persuades both their domestic following and international allies that they are the true victim in the conflict. Three survey studies were conducted to assess the perceptions of citizens of a third-party observer nation (Canada) in relation to two nations in conflict that differ in their historical persecution, namely the U.S. and Israel. Perceptions of the vulnerability of their safety and survival, and their strength to protect themselves against their opponents were hypothesized to mediate differences in the perceived justification for each nation’s conflict actions. Study 1 (*N* = 91) supported this mediational model, with the U.S. seen as less vulnerable and more powerful than Israel, and perceptions of vulnerability accounting for differences in the justifiability of their respective conflict actions. Study 2 (*N* = 315) further demonstrated a moderating effect of Canadians’ shared identity with the nations in conflict; only at lower levels of a shared identity was Israel perceived to be more vulnerable and the mediated relation with the perceived justifiability of its conflict actions retained. Study 3 was conducted 10 years later (2018), administering measures to an independent sample of Canadian participants (*N* = 300). Canadians were found to be significantly less likely to share a common identity with Americans than previously; once again, the mediating role of the perceived vulnerability of the nations in conflict and the justifiability of their actions was conditional on shared identification. The findings contribute to understanding influences on the credibility of victim claims by nations in conflict, as well as implications for how their actions are construed by citizens of a third-party observer nation.

## Introduction

In Western cultures, there is a predilection to support the underdog in conflict situations [1–3]. It has been suggested that this response emanates from principles of fairness and social justice [4]. Alternatively, individuals might otherwise be motivated to bask in the glory of a powerful ally [5], or to maintain a stable status hierarchy [6]. Yet, it is not always clear who constitutes the underdog, as this varies with context, as do perceptions of the legitimacy of the actions taken by the underdog. On the world stage, political leaders play on these factors in the narratives that they spin regarding their involvement in international conflicts [7]. By doing so, leaders attempt not only to solicit greater domestic support, but to garner approval on the world stage, together with moral, if not tangible, support [8]. In this regard, it might be that different strategies are effective depending on the historical underdog (vs. top dog) status of a nation in conflict.

National leaders have, indeed, opted for different strategies to render their conflict actions morally justifiable. For example, President George W. Bush sought to elicit domestic and international support by depicting America as engaging in a ‘war on terror’ [9] and fighting an ‘axis of evil’ [10] in reference to Iraq, Iran, and North Korea. Such a strategy aimed at rendering actions justifiable by raising fears about the magnitude of the threat and violence that could be faced. Over a decade later, amidst his repeated complaints about America being ‘ripped off’ by its allies (and competitors) around the world [11], President Donald J. Trump’s ‘America-First’ campaign conveyed a confident anticipation of triumph in the face of adversities. Both discourses highlight a claim to the collective victimization of the United States, despite its long-standing international status as a ‘superpower’. By contrast, a nation such as Israel, whose foundation was built on addressing centuries of Jewish persecution [12], attempted to gain international support by presenting its conflict actions as necessary to protect shared values, as stated by Israeli Prime Minister Netanyahu, “We stand together to defend democracy” [13–15]. In effect, nations in conflict, whether they are the alleged underdog or top dog, might seek support in the context of their collective victimization at the same time as asserting shared goals and a capacity to contribute to the positive status of other ‘like-minded’ nations. The goal of the present investigation was to assess the perceptions of citizens from a third-party observer nation concerning the vulnerability versus strength of nations in conflict, and the implications of these perceptions for observers’ beliefs about the legitimacy of their conflict actions. Further assessed was the extent to which these relations were moderated by whether the perceived values of the nation in conflict were seen to be shared with those of the observer nation. Changing times certainly may alter these perceptions, and thus we evaluated observer perspectives at distinct historical time points 10 years apart.

### Historical victimization and national vulnerability

There is a tendency to support the most vulnerable in conflict situations [1,3]. The sympathy associated with the continued suffering or fear experienced by a vulnerable group promotes perceptions that protective actions taken by the group are morally justified [1,7,16]. In effect, status as an undeserving victim of illegitimate harm confers moral credentials, and a right to expect symbolic or material reparations [1,17]. While long-standing historical persecution of a group is likely to elicit perceptions of vulnerability [18], more powerful groups also might present themselves as the real victims in a conflict in order to establish moral entitlement and moral outrage [8,19]. In so doing, there is solidification of the ingroup’s identity in recognition of a shared fate and the need for solidarity to ward off threats. In addition, observer groups, including the international community, are more likely to lend support to the perceived victim of conflict [1].

Even as nations in conflict vie for being regarded as the truly victimized, the credibility of such a claim can vary. Although differential perceptions might exist concerning the underdog and top dog opponents in a specific conflict, there is little research to elucidate when a top dog’s claim to victimization is perceived as legitimate. On the one hand, it has been suggested that national strength and power to contend with a threat do not undermine the capacity to establish one’s own group as a victim [8]. On the other hand, a long history of persecution and historical victimization (or lack thereof) might well contribute to the viability of a victim narrative. For example, in contrast to the U.S., which is typically regarded as an economic and military superpower, Israel’s national identity is often perceived as being founded in historical victimization, represented through persecution of Jews through the centuries, including the most recent genocide perpetrated in the Holocaust [1,12,20]. Thus, while both nations might be regarded as top dog in the particular conflicts in which they are currently engaged, among other factors, Israel’s links to historical victimization might render it more credible as a nation engaged in acts that are necessary to ensure its very survival.

### National strength and achieving triumph in the face of adversity

At the same time as observers might sympathize with the underdog, they are inspired by triumph [4]. Indeed, it is notable that U.S. President Trump’s refrain to ‘make America great again’, both implicitly and explicitly in the accompanying rhetoric, highlights America as victorious as it comes out of a period of victimization, unjust treatment and disadvantaged status on the world stage. Accordingly, national strength and pride will have been deservedly earned.

In contrast to depictions of vulnerability, when a group’s strengths are salient, the probability of being perceived as equal and treated with mutual respect increases [4,21]. When a group is regarded as powerful, actions that are taken that could be construed as unjust are seen as more normative, and intervention from outsiders as less likely to be necessary or effective in changing the situation [22]. In essence, people may ‘shift standards’ in how they evaluate injustice, with a bias that favors the more powerful [22,23].

This said, recognizing the strengths of a group also elicits expectations of moral responsibility and obligation. Indeed, when members of a group were subjected to harm in the past (e.g., the Holocaust), and they persevered to overcome their suffering, observers expected them to have learned from the experience [24] and, as a result, be better, stronger people, and they were held to a particularly high standard of moral conduct [16,24,25]. Thus, when a nation in conflict is regarded as strong or triumphant, it may risk observers’ condemnation of its actions against an opponent.

### The present research

The goal of the present research was to determine whether citizens from a nation not directly involved in an international conflict were differentially influenced by perceptions of the vulnerability or strength of the nations in conflict, and the implications for the perceived justifiability of their conflict actions. Three studies were conducted to assess Canadians’ perceptions of the conflict actions of the U.S. and Israel. In particular, the U.S. engaged in a war with Iraq following the September 11, 2001 attacks on the World Trade Towers and the Pentagon by the terrorist organization al-Qaida. The American government claimed that Iraq was harbouring weapons of mass destruction, making it a threat to Western countries, including the U.S. The allied invasion began in March 2003, with a further surge of U.S. troops in 2007; all U.S. combat troops withdrew by December 2011. For decades, Israel has been engaged in an ongoing conflict with Palestinians within the West Bank and Gaza, a struggle that is at the core of the Arab-Israeli conflict. Israel maintained that the Palestinians and their Middle Eastern allies were Israel’s sworn enemies who were intent on eradicating the country and people, and thus represented an existential threat.

Canada was a political ally to both nations, but was not involved in the conflicts being considered. It was expected in Study 1 that differential perceptions of the vulnerability of the security and safety of these nations, as well as their strength to contend with the threat would mediate perceptions of the moral justifiability of their conflict actions. Study 2 assessed the moderating role of Canadians’ shared identity with each nation. Studies 1 and 2 were conducted in 2007 and 2009, respectively, in the era of U.S. President George W. Bush. Study 3 assessed these same perceptions in 2018 when the U.S. was under the presidency of Donald J. Trump. Thus, we were able to evaluate whether changes in the priorities of U.S. domestic and international policies altered the factors contributing to Canadians’ perceptions of the moral justifiability of conflict actions.

## Study 1

The credibility of a nation claiming victim status varies not only with the historical persecution of its peoples, but as well as a function of perceived size relative to its opponent, the resources at its disposal, the aggressiveness of the actions taken, and whether the conflict is viewed as deserved or brought upon itself [3,26]. These elements need to be balanced so that the nation in conflict is not itself perceived as the top dog, but rather as the victim of an enemy that is evil and of sufficient strength that the nation’s survival is at risk [1,27,28]. In Study 1, it was hypothesized that compared to Israel, the U.S. would be perceived as lower in the vulnerability of its safety and survival, and higher in its strength to confront its adversaries successfully, and these perceptions would mediate differences in the perceived justifiability of conflict actions.

### Materials and methods

From March to August 2007, first year university students at a Canadian university (*N* = 91; female *n* = 57; male *n* = 34) ranging from 18 to 54 years of age (*M* = 22.6; *SD* = 6.58 years) participated in an online study concerning perceptions of international conflicts. The majority of students reported their ethnicity to be White (75.8%), with the remainder identifying as Black (2.2%), Asian (14.6%), or other (7.4%). Students who self-reported being Jewish, Muslim or of Middle Eastern or American origins were not included in the analyses, as they may be less likely to perceive themselves simply as observers of the conflicts.

After providing informed consent, participants were randomly assigned to complete one of two versions of a questionnaire. The questions were substantively the same, but were modified, where relevant, to either refer to the Israeli/Palestinian (*n* = 46) or U.S./Iraq conflicts (*n* = 45). Upon completion, participants were debriefed and awarded course credit for their participation. All procedures were approved by the Carleton University Research Ethics Board-B (#108382).

#### Nations’ vulnerability

Perceptions of the extent to which the safety and survival of each nation in conflict was threatened was assessed using three items, including “Full American withdrawal from Iraq would threaten the safety and survival of the USA”, “If the USA did not actively fight against terrorism, its own safety and survival would be threatened”, and “The USA has become highly vulnerable to terrorist acts on its home ground”. The modified items for the Israel/Palestinian conflict were “Full recognition of Palestinian demands would threaten the safety and survival of Israel”, “If Israel did not actively fight against terrorism, its own safety and survival would be threatened”, and “Israel’s position in the Middle East is highly vulnerable.” Responses were on 7-point scales, ranging from 1 ‘disagree strongly’ to 7 ‘agree strongly’, and were averaged to provide an index of perceived vulnerability (Cronbach’s alpha = .74). It is noted that the items assessing perceptions of the vulnerability of the two nations were not identical, reflecting differences in specific features of the respective conflicts. Importantly, when the single item in common was analysed, the pattern of findings was identical. Thus, results using the 3-item measure were reported, as findings based on multi-item measures may be more robust across contexts.

#### Nations’ strength

Perceptions of the strength of the two nations in conflict were assessed using two items, including “The USA/Israel has sufficient military might to protect itself against a threat from any other nation(s) in the Middle East” and “The USA/Israel has become a relatively powerful nation that, realistically, does not have to be concerned about its security.” These items were rated on 7-point scales, ranging from 1 ‘disagree strongly’ to 7 ‘agree strongly’, and were averaged to provide an index of perceived strength, (*r* = .31, *p* = .003).

#### Justifiability of conflict actions

Participants indicated their agreement with each of five statements, including for example, “The Iraqis should be held accountable for American actions to protect itself against terrorist violence” and “Israel is justified in its harmful actions against the Palestinians”, on a 7-point rating scale ranging from 1 ‘strongly disagree’ to 7 ‘strongly agree’. Identical items were used for the two nations in conflict, modifying the target groups being referred to (Cronbach’s alpha = .80).

### Results and discussion

#### Differences in perceptions of U.S./Iraq and Israel/Palestinian conflicts

Analyses of variance (ANOVAs) conducted to assess differences in perceptions of the two nations in conflict indicated that, as expected, Israel was perceived to be more vulnerable and less powerful than the U.S. (Table 1). Moreover, in line with research demonstrating that the actions of underdogs, especially those that continue to be victimized, are more likely to be perceived as morally legitimate [16,17], participants regarded Israel’s conflict actions as more justified than those of the U.S.

**Table 1:** Perceptions of the Israeli-Palestinian vs. U.S.- Iraq Conflicts in Study 1.

	Israel—Palestinian		U.S.—Iraq		
	Mean	*SD*	Mean	*SD*	eta^2^
Perceived vulnerability	4.92	1.17	3.46	1.37	.251***
Perceived strength	3.24	1.16	4.22	1.23	.146***
Justifiability of actions	4.08	0.91	3.11	1.30	.159**

Mean scores could range from 1 to 7.

** *p* < .01

*** *p* < .001

Although it might have been expected that perceptions of vulnerability and strength would be inversely correlated, the partial correlation (controlling for nation) between them was not significant, *partial r* = -.08, *p* = .486. Thus, a nation can be vulnerable to its enemies, but still be perceived to have the power to defeat them.

#### Mediated relations between nations in conflict and perceived justifiability of actions

**Fig 1:**
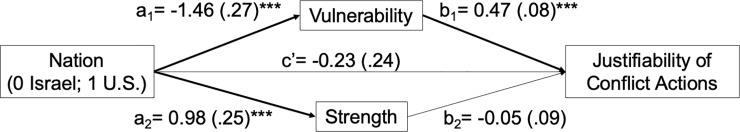
Unstandardized path coefficients (standard errors) of model assessing the mediating roles of perceptions of vulnerability and strength in the relation between nation in conflict and perceived justifiability of actions (Study 1). *** *p* < .001. a_1_b_1_ = Indirect effect of nation in conflict on action justifiability through perceived vulnerability; a_2_b_2_ = Indirect effect of nation in conflict on action justifiability through perceived strength; c' = Direct effect of nation in conflict on action justifiability.

To assess whether the differences in the perceived justifiability of the actions of the two nations (with Israel coded 0 and the U.S. coded 1) were mediated by perceptions of vulnerability and strength, the PROCESS macro (version 3.3) was used, applying the mediation model 4 [29]. The macro was set to use bootstrapping procedures with 5000 resamples. The full model that included the nations in conflict and the two mediating variables was significant, *R*^2^ = .404, *F*(3,86) = 19.44, *p* < .001. The direct path between nation in conflict and action justifiability was not significant, *b* = -0.23, *se* = 0.25, CI_.95_[-0.72, 0.25]. The 95% confidence intervals for each mediator indicated that, as seen in Fig 1, this relation was mediated by perceived vulnerability, Effect = -0.69, *se* = 0.17, CI_.95_[-1.04, -0.38], but not by national strength, Effect = -0.05, *se* = 0.10, CI_.95_[-0.27, 0.15].

In short, as expected, Israel, whose Jewish people have been historically victimized, was seen as being more vulnerable in terms of its safety and survival, and as having less strength to protect itself than the U.S. Notably, the items assessing perceptions of vulnerability differed to reflect the contextual features of the conflicts in question, and hence such perceptions might have reflected differences in the items. However, even when only the item in common (“If the U.S./Israel did not actively fight against terrorism, its own safety and survival would be threatened”) was analysed, the same pattern of results was evident. Perceived vulnerability, but not the strength of a nation, accounted for further differences in perceptions of the justifiability of the conflict actions. Thus, it appears that factors that enhance a nation’s claim to victimization may play a stronger role in how citizens of an observer nation regard their conflict actions [8] than does the perceived capacity of a nation to protect itself from its enemies.

## Study 2

If an observer identifies with the struggles of a victimized group, as a matter of social justice there is a propensity to sympathize with, and root for, the underdog [4]. Hence, while continuing to emphasize its collective victimization and vulnerability, nations in conflict may attempt to tie their narrative to the values and priorities of others in the international community in an effort to strengthen observers’ solidarity and moral backing [19]. In this regard, Israel’s identification with the ‘war for democracy’ was a strategy for eliciting a shared identity with observer nations, thereby giving licence for taking protective actions [28,30], and consolidating perceptions that conflict actions were necessary and legitimate [31]. In effect, observers who perceive a ‘common moral community’, particularly in relation to a historically victimized nation, may be especially sensitive to its continued vulnerability, and the need for support [32]. In contrast, in the absence of such a sense of shared identity, observers might distance themselves, even from those who have been historically victimized (e.g., psychological numbing [33]), and as a result be less likely to acknowledge or empathize with a nation’s vulnerability, thereby diminishing the extent to which their conflict actions are regarded as legitimate [34].

However, receiving a shared identity could also confer a greater perception of national strength and the resources to contend with identity threats. Such identification with a well-positioned ingroup might enhance perceptions of a nation’s capacity to protect itself. If so, it is possible that the greater perceived strength of a nation in conflict that is derived from observers’ shared identity could trigger a sanctioning process, wherein the nation in conflict is subsequently held to an especially high standard of moral conduct, with expectations of restraint in its actions toward an opponent [28,35]. In this instance, citizens of an observer nation may be motivated by the need to maintain the moral integrity of the shared ingroup identity [35,36], and as a result regard conflict actions as less justified. Thus, it was hypothesized that the mediating role of the perceived vulnerability and strength of the nations in conflict on perceptions on the justifiability of their actions would be conditional on levels of shared identification.

### Method and materials

From March through May 2009, 315 community participants were recruited through posters and advertisements on different Canadian websites inviting them to take part in an online study concerning perceptions of international conflicts. The majority of participants were female (*n* = 215; male *n* = 100), ranging from 18 to 66 years of age (*M* = 33.17; *SD* = 10.75 years), and reported their ethnicity to be White (73.0%), with the remainder Black (1.6%), and Asian (18.8%), or other (6.6%). As in Study 1, participants who self-reported being Jewish, Muslim or of Middle Eastern or American origins were not included in the analyses.

After providing informed consent, participants were randomly assigned to complete one of two versions of the questionnaire, which either referred to the Israeli/Palestinian (*n* = 156) or U.S./Iraq conflict (*n* = 159). Upon completion of the study, participants received an online debriefing along with a $10 gift certificate for their participation.

As in Study 1, measures of the perceived vulnerability (Cronbach’s alpha = .63) and strength (*r* = .44, *p* < .001) of the two nations, and the justifiability of their actions (Cronbach’s alpha = .81) were included. Shared identity with the target nations was assessed by asking participants to rate Israel and the U.S. (among a list of 15 other nations) in terms of their perceptions that each shared common values and priorities with Canadians, using a 7-point scale ranging from 1 ‘nothing at all in common’ to 7 ‘a lot in common’. In addition, the extent to which Israel and the U.S. (along with a list of 11 other nations) was perceived to be “typical of what it means to be a free and democratic nation” was rated using a 7-point scale ranging from 1 ‘not at all typical’ to 7 ‘extremely typical’. It is recognized that ascribing to democratic values is only one of many possible features of a shared national identity, and may not be a feature that is important to Canadians’ identification with all national identities. However, in the context of international conflicts in which this rhetoric has been made salient, as both the U.S. and Israel had done (i.e., the ‘war on democracy’), it was thought to be an important aspect of shared identification. Indeed, responses to these two items were highly correlated, *r* = .67, *p* < .001, and so they were combined to create a single index of shared identity. Importantly, the pattern of results remained the same whether a one- or two-item index of shared identity was used.

### Results and discussion

#### Differences in perceptions of U.S./Iraq and Israel/Palestinian conflicts

ANOVAs conducted to assess differences in perceptions of the two nations indicated that, as in Study 1, Israel was perceived to be more vulnerable and have less strength to protect itself than the U.S. (Table 2). Perhaps the greatest difference in perceptions was the extent to which the two nations were perceived to share a common identity with Canadians, in that Americans were considered to be much more aligned with Canadian identity. Nonetheless, participants were more likely to perceive Israel’s conflict actions as more justifiable than those of the U.S.

**Table 2:** Perceptions of the Israeli-Palestinian vs. U.S.- Iraq Conflicts in Study 2.

	Israel—Palestinian		U.S.—Iraq		
	Mean	*SD*	Mean	*SD*	eta^2^
Shared identity	3.15	1.32	5.81	0.95	.572***
Perceived vulnerability	4.72	1.15	3.78	1.25	.126***
Perceived strength	3.41	1.11	4.23	1.32	.101***
Justifiability of actions	4.03	1.14	3.57	1.17	.038**

Mean scores could range from 1 to 7.

** *p* < .01

*** *p* < .001

Perceptions of vulnerability and strength (controlling for nation) were negatively correlated, *partial r* = -.29, *p* < .001. The significance of this relation, which was absent in Study 1, may be due to the greater variability emanating from a community sample. Although both lower perceived vulnerability, *r* = -.27, *p* < .001, and greater strength, *r* = .25, *p* < .001, were associated with a shared identity, neither of these relations was significant when controlling for nation, *partial r*s = .02. Shared identity was not directly related to perceptions of the justifiability of conflict actions, *r* = -.02, *p* = .734.

#### Conditional mediated model as a function of shared identity

To assess whether the mediated pathways between the nations in conflict and the perceived justifiability of their actions depended on observers’ shared identity, a conditional mediation analysis was conducted. To do so, the PROCESS macro (version 3.3) was used, applying model 7 [29], which assessed whether the moderating variable (shared identity) attenuated or increased differences in the relation between the predictor (nation in conflict, with Israel coded 0 and the U.S. coded 1) and mediators (perceived vulnerability and strength), thereby influencing differences in the perceived justifiability of their respective conflict actions. The macro was set to use using bootstrapping procedures with 5000 resamples. Variables were mean centred for the calculation of the cross-products (interactions).

**Fig 2:**
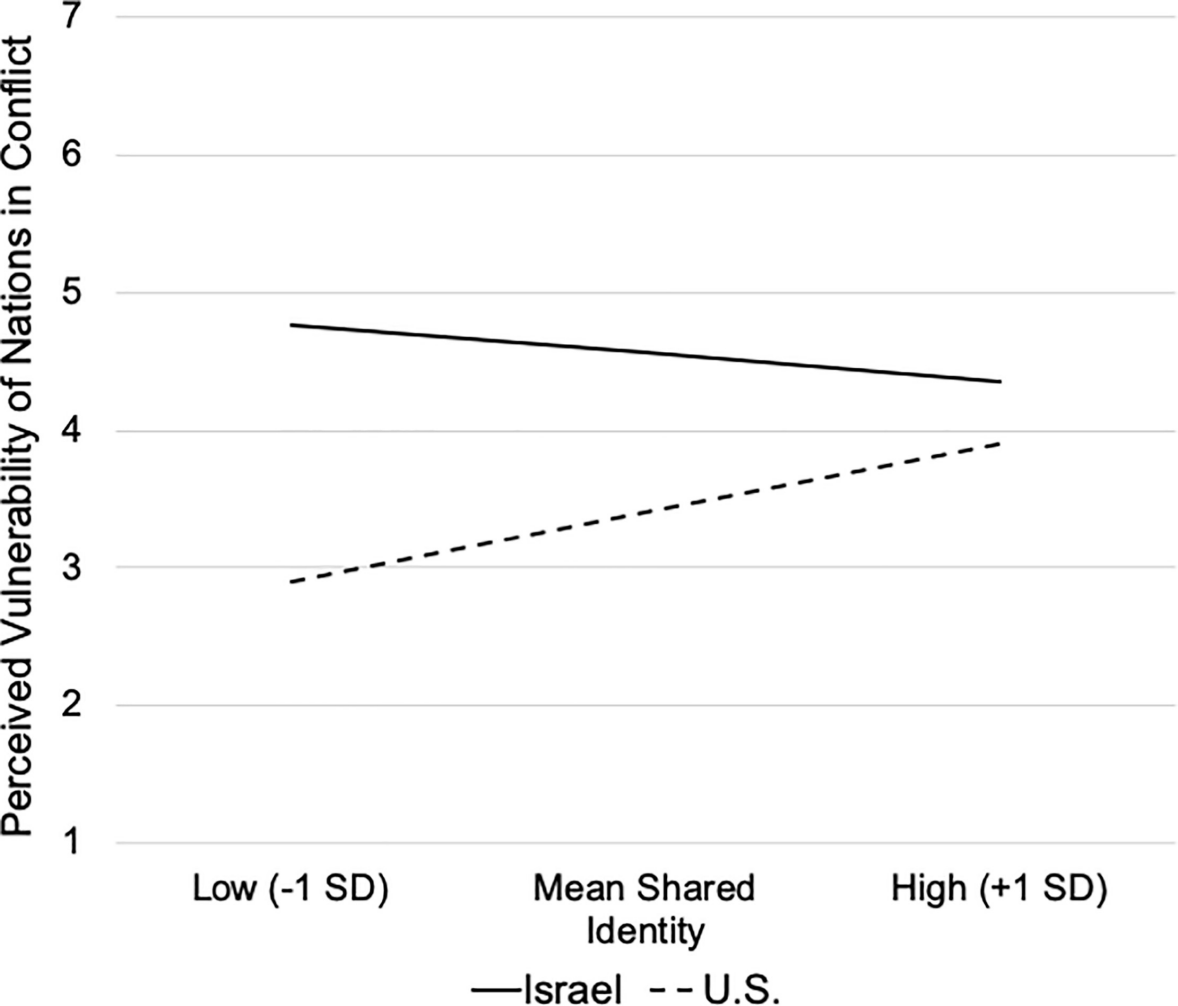
Simple Slopes of the relations between shared identity and perceived vulnerability of Israel versus the U.S.

As seen in Table 3, shared identity significantly moderated differences in perceptions of the vulnerability, *R*^2^ = .029, *F*(1,311) = 10.81, *p* = .001, but not strength of the two nations, *R*^2^ = .001, *F*(1,311) = 0.20, *p* = .654. Simple slope analyses conducted at 1 *SD* above and below the mean ratings of shared identity (Fig 2) indicated that at lower levels of shared identification with the nation in conflict, differences in their perceived vulnerability were exacerbated, *b* = -1.87, *se* = 0.33, *p* < .001; with increasingly high levels of shared identification, differences in the perceived vulnerability of the U.S. and Israel dissipated, *b* = -0.45, *se* = 0.26, *p* = .088. This pattern was primarily accounted for by the positive relation between shared identification with the U.S. and perceptions of the vulnerability, *b* = 0.29 (*se* = .10), *p* = .004, that was absent with Israel, *b* = -0.12 (*se* = .07), *p* = .10.

**Table 3:** Unstandardized Coefficients of Model Assessing the Moderating Role of Shared Identity on the Mediated Relations between Nation in Conflict and Perceived Justifiability of Actions (Study 2).

Effect	Coefficient	*se*	Lower CI_.95_	Upper CI_.95_
Mediator 1 = Vulnerability				
Nation	-1.16	0.21	-1.57	-0.74
Shared identity	0.09	0.06	-0.04	0.21
Nation*identity	0.40	0.12	0.16	0.64
Mediator 2 = Strength				
Nation	0.75	0.22	0.32	1.18
Shared identity	0.03	0.06	-0.10	0.15
Nation*identity	0.06	0.13	-0.19	0.31
Outcome (Y) = Action justifiability				
Nation	-0.14	0.13	-0.39	0.11
Vulnerability	0.47	0.05	0.37	0.57
Strength	0.14	0.05	0.05	0.24

Note: Coefficients represent the effects of the predictor (nation in conflict: 0 = Israel, 1 = U.S.) and moderator (shared identity) variables and their interaction on each of the mediation variables (perceived vulnerability and strength of the nations), followed by the unique effects of the predictor and mediating variables on the outcome variable (justifiability of conflict actions).

As hypothesized, the mediating role of perceived vulnerability in the prediction of the moral justifiability of the conflict actions was conditional on levels of shared identity, Index = 0.19, CI_.95_[0.07, 0.33]. Contrary to expectations, the mediating role of vulnerability was greatest when the nation in conflict was not seen to share a common identity with Canadians, Effect = -0.88, *se* = 0.21, CI_.95_[-1.30, -0.51], whereas mediation was no longer evident when nations were strongly perceived to share an identity, Effect = -0.21, *se* = 0.14, CI_.95_[-0.49, 0.05]. The absence of a significant mediating role of the perceived strength of the nations in conflict was not altered by levels of shared identity, Index = 0.01, CI_.95_[-0.04, 0.53]

In sum, as in Study 1, perceptions of Israel and the U.S. differed, with the U.S. being seen as less vulnerable and stronger. Not surprisingly, participants indicated that Canadians shared a stronger identity with the U.S than with Israel. This shared identity was not directly related to the perceived justifiability of conflict actions, possibly because identification in itself might elicit multiple competing psychosocial motivations. For example, observers who perceive a strong shared identity might be motivated to enhance the ingroup identity by justifying conflict actions, or conversely, experience greater collective guilt for perpetrating harm [37] or hold higher expectations of restraint to maintain the moral integrity of the identity [35,36]. However, in the absence of a shared identity, participants were more likely to regard Israel as vulnerable (relative to the U.S.), and under these conditions, vulnerability was an especially strong mediator of the differences in the perceived justifiability of each nations’ actions. Although not predicted, this latter finding is in line with research suggesting that support for the underdog might be abandoned when the context is perceived to be self-relevant, and particularly when the consequences associated with the outcomes are high [2]. Similarly, pre-existing affinities may serve to over-ride processes that would elicit support for the underdog in favor of ingroup biases [38]. The present study did not include measures of self-relevance or personal affinities, but it is possible that shared identification similarly served to diminish the salience of factors that would otherwise evoke underdog support. In contrast, when participants had no vested identity interest in the conflict, they perceived the nation that had a long history of victimization (Israel) as most vulnerable, and hence more justified in their actions.

## Study 3

The rhetoric of the U.S. appeared to shift under the presidency of Donald J. Trump. Although the U.S. continued to assert a narrative of collective victimization, it also highlighted triumph in the face of such adversity (‘make America *great* again’). Although this message might have been intended for a domestic audience, it was nonetheless broadcast around the world. In addition, international policies (including economic agreements, the U.S. position on fossil fuels and climate change, military decision-making, etc.) isolated the U.S. from allies with which it had historically shared a common identity, including Canada. Thus, it was expected in Study 3 that a sense of shared identity would be weaker. Ironically, based on the findings of Studies 1 and 2, this is precisely when a nation’s perceived vulnerability would be a strong predictor of observers’ judgements of the moral legitimacy of its conflict actions.

Study 3 sought to assess the predictors of Canadians’ perceptions of the justifiability of the actions of the U.S. and Israel under these changed conditions. Although the specific disputes between Israel, the Palestinians, and the regional Arab nations were different, on the whole, Israel’s conflict situation remained the same. In Iraq, at the invitation of the Iraqi government, in 2014, the U.S. launched a military intervention to combat the Islamic State of Iraq and the Levant (ISIL). The U.S. maintained an active role in this civil war, but in February 2018, announced that it would begin to withdraw troops from Iraq. In addition to these conflicts, in Study 3, Canadians were asked about their perceptions of the increasingly hostile relationship between the U.S. and North Korea. The tension between the U.S. and North Korea had been present for decades, particularly in relation to the North Korean development of nuclear missiles. Successive U.S. presidents either failed to reach an agreement with North Korea, or agreements were not honoured. At the time of conducting this study, the U.S. was sending strong and provocative signals to North Korea, and the possibility of a summit between President Trump and North Korean Supreme Leader Kim Jung-un had *not* yet been announced. Thus, the U.S. conflict with North Korea was of a diplomatic rather than military nature.

### Method and materials

In January through February 2018, participants were recruited using Amazon MTurk, with access to the survey restricted to Canadian citizens (*N* = 300; 179 males, 115 females, 6 other gender) greater than 18 years of age (*M* = 32.06; *SD* = 9.64 years). The majority of participants reported their ethnicity to be White (71.8%), with the remainder Black (4.0%), and Asian (13.9%), or other (10.3%). After providing informed consent, participants were randomly assigned to complete one of three versions of the questionnaire, which either referred to the Israeli/Palestinian (*n* = 94), U.S./Iraq conflicts (*n* = 91), or the conflict between the U.S. and North Korea (*n* = 115). Participants who self-reported being Jewish, Muslim, or of Middle-Eastern, American, or North Korean origins were assigned to survey conditions that were not directly associated with their religious or national background. Upon completion of the study, participants received an online debriefing along with $3USD MTurk payment. Measures of the perceived vulnerability (Cronbach’s alpha = .65) and strength (*r* = .42, *p* < .001) of the U.S. and Israel, the justifiability of their conflict actions (Cronbach’s alpha = .79), and Canadians’ sense of shared identity (*r* = .47, *p* < .001) were identical to those used in Studies 1 and 2, with modification, where appropriate, to be inclusive of reactions to the U.S. actions toward North Korea. Specifically, the perceived vulnerability item “Full American withdrawal from countries targeted by terrorists would threaten the safety and survival of the USA” was replaced with “American aggression toward its enemies would threaten the safety and survival of the USA”. In addition, items making reference to Iraq were replaced with North Korea.

### Results and discussion

#### Differences in perceptions of the nations in conflict

ANOVAs conducted to assess differences in perceptions of the three intergroup conflicts indicated that, as in the previous studies, Israel was perceived to be more vulnerable than the U.S., to have less power to protect itself, and to share less of a common identity with Canadians (Table 4). In addition, participants were more likely to perceive Israel’s actions to be more justified compared to those of the U.S. in Iraq, but not those of the U.S. toward North Korea, perhaps because the latter had not reached a point of military aggression, or perhaps because North Korea represented a greater threat to the U.S. and its allies.

**Table 4:** Perceptions of the Israel vs. U.S. Conflicts in Study 3 (2018).

	Israel-Palestinian		U.S.—Iraq		U.S.—North Korea		
	Mean	*SD*	Mean	*SD*	Mean	SD	eta^2^
Shared identity	3.38 _a_	1.23	5.44 _b_	1.13	5.45 _b_	1.18	.397***
Perceived vulnerability	4.41 _a_	1.28	3.87 _b_	1.26	3.75 _b_	1.45	.043***
Perceived strength	3.90 _a_	1.24	4.61 _b_	1.43	4.88 _b_	1.39	.086***
Justifiability of actions	3.72 _a_	1.24	3.30 _b_	1.27	3.90 _a_	1.25	.039**

Mean scores could range from 1 to 7. Columns with different subscripts differed at *p* < .05.

** *p* < .01

*** *p* < .001

Partial correlations (controlling nation in conflict) indicated that, just as in Study 2, perceptions of national vulnerability and strength were negatively correlated, *partial r* = -.18, *p* = .002. In addition, shared identity was associated with perceptions of strength, *r* = .30, *p* < .001, and the justifiability of conflict actions, *r* = .15, *p* = .011, but was not directly related to perceived vulnerability, *r* = -.01, *p* = .804.

To assess changes over time, only responses to the conflicts that were assessed in both 2009 and 2018 were compared, namely the Israeli-Palestinian and U.S.-Iraq conflicts. As hypothesized, in a 2 (time) x 2 (nation in conflict) between-groups ANOVA, the interaction in relation to a shared identity with the nation in conflict was significant, *F*(1,494) = 7.95, *p* = .005, eta^2^ = .016. In particular, as seen in Tables 2 and 4, although there were no differences over time in Canadians’ identification with Israel, in 2018, Canadians reported a weaker shared identity with the U.S. It is possible that the policy shifts of the U.S. in relation to its allies were linked to these differences over time. In addition, main effects for time were found, in that perceptions of the strength of both Israel and the U.S. to protect their interests increased over the 10 years, *F*(1,494) = 12.35, *p* < .001, eta^2^ = .024, and the perceived justifiability of their conflict actions decreased, *F*(1,494) = 7.05, *p* = .008, eta^2^ = .014. Although these effect sizes were small, this pattern of change over time is in keeping with increasingly vocal global opposition to both Israel’s and the U.S.’s intervention in the Middle East. It is also in line with the hypothesized relationship between the perceived strength of a nation and evaluations of the justifiability of its conflict actions [24,28].

#### Conditional mediation model as a function of shared identity

To assess whether the mediated model predicting the perceived justifiability of the conflict actions of the nations was moderated by shared identity with Israel versus the U.S., a conditional mediation analysis (PROCESS model 7) was conducted. National conflict was treated as a multi-categorical variable, wherein Helmert coding was employed. Comparison 1 (X1) assessed differences between the Israel-Palestinian conflict and the mean of the two U.S. conflicts; X2 compared responses to the U.S. in the context of the Iraq vs. North Korean conflicts.

**Fig 3:**
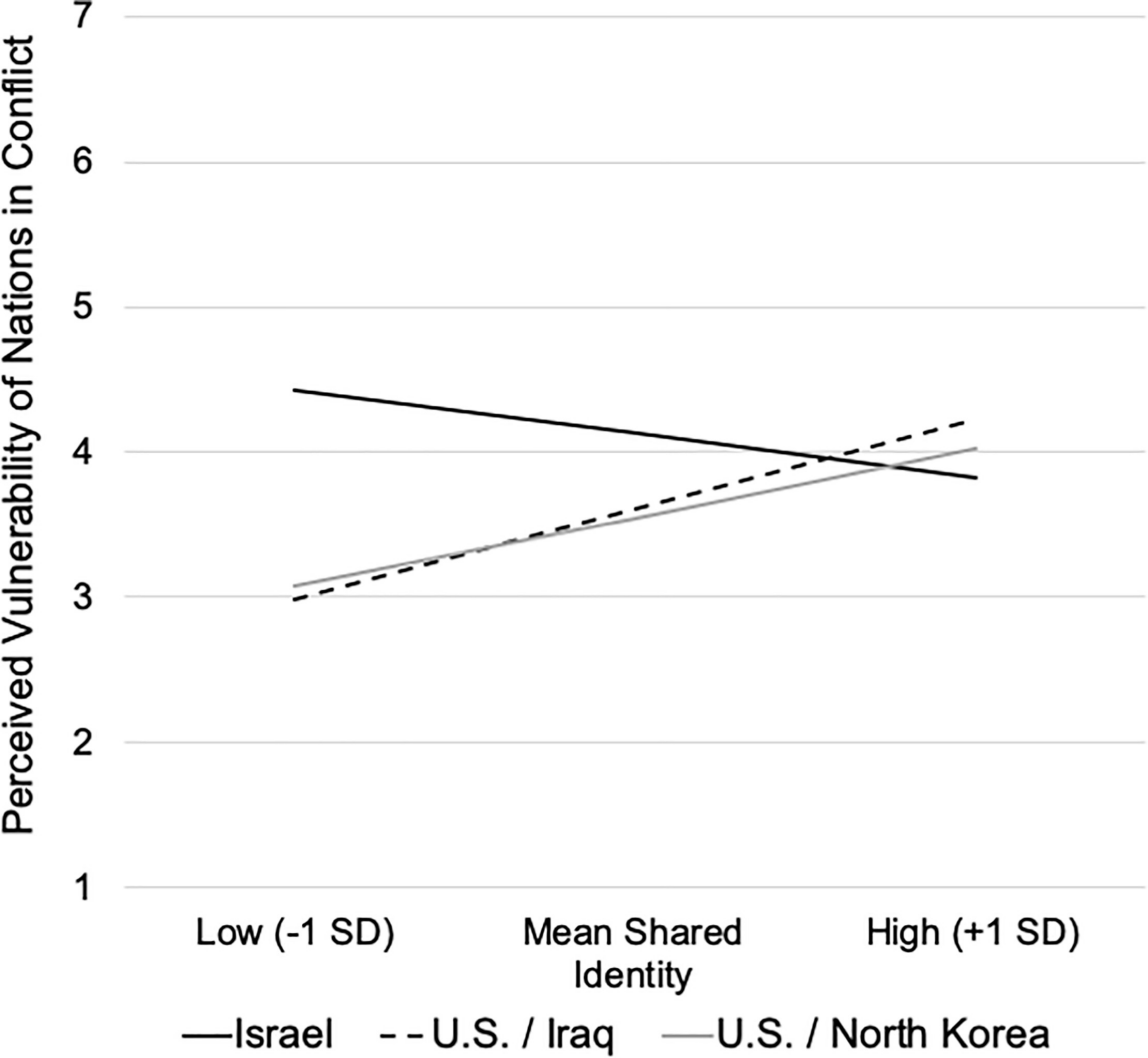
Simple Slopes of the relations between shared identity and perceived vulnerability of Israel versus the U.S. in the Iraq and North Korean conflicts.

As reported in Table 5, a shared identity moderated the relation between nations in conflict and their perceived vulnerability, *R*^2^ = .052, *F*(2,292) = 8.60, *p* < .001, and in particular moderated the difference in the perceived vulnerability of Israel and the U.S. (X1), rather than variations in perceptions of the vulnerability of the U.S. in the different conflict arenas (X2). Specifically, simple slope analyses indicated that, as in Study 2, differences in the perceived vulnerability of the two nations were most evident at lower levels of shared identification with the nations in conflict, *b* = -1.40, *se* = 0.24, *p* < .001; at higher levels of shared identification there was no difference in the perceived vulnerability of Israel and the U.S., *b* = 0.30, *se* = 0.37, *p* = .412 (Fig 3). As in Study 2, this pattern was primarily accounted for by the positive relation between perceptions of the vulnerability and shared identification with the U.S, whether the conflict involved either Iraq, *b* = 0.41 (*se* = .12), *p* = .001, or North Korea, *b* = 0.32 (*se* = .10), *p* = .003, a relation that was largely absent with respect to Israel, *b* = -0.20 (*se* = .10), *p* = .057.

**Table 5:** Unstandardized Coefficients of Model Assessing Moderating Role of Shared Identity on the Mediated Relations between Nation in Conflict and Perceived Justifiability of Actions (Study 3).

Effect	Coefficient	se	Lower CI_.95_	Upper CI_.95_
Mediator 1 = Vulnerability				
Nation				
X1 Israel vs. US	-0.55	0.23	-1.00	-0.10
X2 US: Iraq vs. N. Korea	-0.06	0.21	-0.45	0.35
Shared identity	0.18	0.06	0.05	0.30
Nation * Identity				
X1 Israel vs. US	0.56	0.14	0.29	0.83
X2 US: Iraq vs. N. Korea	-0.10	0.16	-0.41	0.22
Mediator = Strength				
Nation				
X1 Israel vs. US	0.39	0.24	-0.08	0.86
X2 US: Iraq vs. N. Korea	0.51	0.22	0.08	0.93
Shared identity	0.21	0.07	0.07	0.34
Nation * Identity				
X1 Israel vs. US	-0.09	0.14	-0.37	0.18
X2 US: Iraq vs. N. Korea	-0.38	0.17	-0.70	-0.05
Outcome (Y) = Action justifiability				
Nation				
X1 Israel vs. US	-0.07	0.15	-0.23	0.36
X2 US: Iraq vs. N. Korea	0.63	0.16	0.32	0.95
Vulnerability	0.42	0.05	0.33	0.52
Strength	0.07	0.05	-0.02	0.17

Note. Coefficients represent the effects the predictor (nation in conflict comprising two Helmert coded contrast variables) and moderator (shared identity) and their cross-products on each of the mediation variables (perceived vulnerability and strength of the nations), followed by the unique effects of the predictor and mediating variables on the outcome variable (justifiability of conflict actions). X1 and X2 are the Helmert contrasts that were entered to assess the effects of nation and the relevant criterion variables.

In addition, perceiving a shared identity moderated the mediating role of perceived vulnerability in accounting for differences in perceptions of the justifiability of the actions of Israel versus the U.S., Index (X1) = 0.24, *se* = 0.07, CI_.95_[.11, .37]. As in Study 2, the mediating role of perceived vulnerability was greatest when observers did not share a common identity with the nation in conflict, Effect = -0.55, *se* = 0.12, CI_.95_[-0.81, -0.32], whereas mediation was no longer evident when they were strongly co-identified, Effect = 0.13, *se* = 0.17, CI_.95_[-0.22, 0.45]. This mediated relation did not account for differences in the perceived justifiability of U.S. actions in Iraq versus North Korea, Effect (X2) = -0.05, *se* = 0.08, CI_.95_[-0.21, 0.11], nor was this mediated relation moderated by shared identity, Index (X2) = -0.04, *se* = 0.07, CI_.95_[-.18, .11]. Thus, it appears that this counter-intuitive finding associated with the greater role of perceived vulnerability when observers do not report a shared identity with the nations in conflict replicated across time and with consideration of another American conflict.

**Fig 4:**
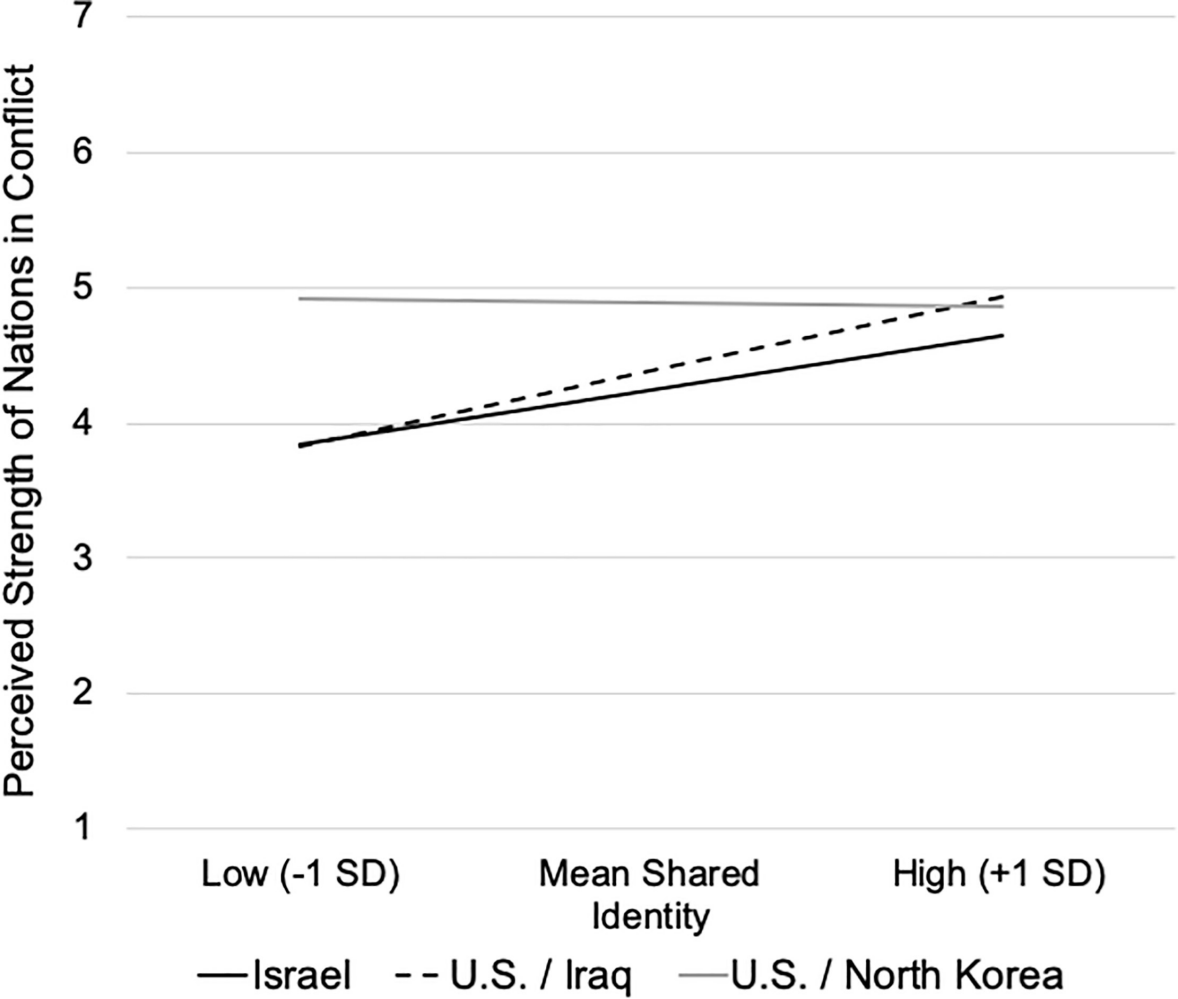
Simple Slopes of the Relations between Shared Identity and Perceived Strength of Israel versus the U.S. in the Iraq and North Korean conflicts.

Uniquely in Study 3, shared identity moderated the relation between nation in conflict and perceptions of national strength, *R*^2^ = .018, *F*(2,292) = 3.04, *p* = .049, accounting for the variations in perceptions of the U.S. in Iraq and North Korea (X2), rather than variations between Israel and the U.S. (X1) (Table 5). Specifically, as shown in Fig 4, when Canadian participants were less inclined to report a shared identity, their perceptions of the strength of the U.S. in North Korea were greater than they were in relation to the U.S. in Iraq, *b* = 1.08, *se* = 0.40, *p* = .008; at higher levels of shared identification there was no difference in the perceived strength of the U.S. in the North Korean conflict, *b* = -0.07, *se* = 0.24, *p* = .781. In effect, while perceptions of U.S. strength in regard to North Korea were independent of sharing a common identity with the U.S., *b* = -0.02 (*se* = .11), *p* = .888, shared identification and perceptions of national strength were positively related when the U.S was in Iraq, *b* = 0.36 (*se* = .13), *p* = .006, and for Israel, *b* = 0.27 (*se* = .11), *p* = .016. Such variations of shared identity did not alter the absence of a mediating role of perceived strength in the differences in the perceived justifiability of actions taken in the two U.S. conflict arenas, Index(X1) = -0.01, *se* = 0.014, CI_.95_[-.04, .01], Index(X2) = -0.03, *se* = 0.03, CI_.95_[-.09, .02].

To summarize, as expected, Canadians’ sense of shared identification with the U.S. (but not Israel) diminished in 2018 relative to 2009. As in Studies 1 and 2, Israel was seen as more vulnerable than the U.S. in terms of its safety and survival, and as having less power to protect itself. In the absence of a shared identity, Canadians regarded Israel as even more vulnerable, which in turn, was associated with greater perceptions of the justifiability of conflict actions. It would appear that, once again, Israel was more likely to garner support for its actions to protect its vulnerable status when observers did *not* have a vested identity interest in the nations in conflict. Moreover, despite the deteriorating relationship between Canada and the U.S., and the diminished sense of shared identity, the pattern of mediated relations predicting the legitimacy Canadians attributed to the conflict actions remained the same across the studies. At the same time, the U.S. was viewed as stronger and its conflict actions as more justified when they involved a diplomatic strategy, as was the case in North Korea. This said, the differential perceptions of U.S. strength across the conflict arenas dissipated when there was a strong shared identity, suggesting that when the identity was self-relevant, other factors contributed to observers’ evaluations of American conflict actions [32].

## General discussion

The findings of the present investigation were consistent with the view that observers of a nation in conflict will be most sympathetic when the nation is perceived to have been victimized, and its safety and survival continues to be tenuous [16,24,25]. In particular, despite the fact that the nations whose actions were evaluated in the present studies varied considerably in their histories and current treatment globally, and that both are often considered top dogs in the conflict arenas in which we placed them (Israel’s actions in relation to the Palestinians; U.S. actions in relation to Iraq/North Korea), the more vulnerable their safety and survival was perceived to be, the greater the legitimacy that was attributed to their actions. Moreover, the greater perceived vulnerability of Israel, whose Jewish population has an entrenched history of persecution, was found to account for differences in how their conflict actions were viewed in comparison to those of the U.S. It seems that status as an underdog, or victim, in a conflict may vary as a function of perceptions of the historical experiences of a group or nation that render victim claims to be more or less credible [12].

In the present study, whether citizens of an observer country viewed the respective nations as sharing common goals and democratic values was a consistent moderator of the differences in how the two nations in conflict were regarded. As expected, Americans were seen as sharing more in common with Canadians than were Israelis. However, a stronger shared identity diminished the extent to which the nations in conflict were differentially regarded in terms of their vulnerability, and attenuated the importance of such vulnerability in rendering perceptions of their actions to be morally justified. This may be counter-intuitive, as it was expected that strong identification with a nation would have provided a motivational basis for empathizing with its vulnerability in the conflict in which it was embedded, and hence, the perceived justifiability of its actions. Indeed, a shared identity and perceptions of U.S. vulnerability were positively related, such that, when there was a strong sense of shared identity, Canadians’ perceptions of the vulnerability of the U.S. were equal to the perceived vulnerability of Israel. As vulnerability was a key predictor of the justifiability of conflict actions, shared identification appears to serve a top dog nation such as the U.S. well. In contrast, by enhancing the underdog’s shared identity with observer nations, concerns about its survival may be reduced, possibly triggering the tendency to hold groups that have been historically victimized to a higher standard of moral behaviour, and to do no harm to others [24,36]. Alternatively, given Canada’s geographical proximity to the U.S., it might be that a strong shared identity reflected a recognition of the common fate that Canadians might experience with the U.S. and hence the need to take protective actions. In this regard, the meaning that is ascribed to the shared identity could itself influence perceptions and actions of the ingroup and observers alike [32]. For example, had the index of identity highlighted shared humanitarianism, rather than being a free and democratic nation, the conditional processes associated with the justifiability of conflict actions might have been different. Finally, it should be noted that the current data are correlational, and so conclusions about the role of a shared identity can only be tentatively suggested.

Although the U.S. and Israel were perceived to differ in their strength to defend themselves from their opponents, these perceptions did not account for differences in observers’ perceptions that conflict actions were less justified. Perceptions of strength and vulnerability were mildly negatively correlated in Studies 2 and 3, but it is clear that they are not simply the inverse of one another. In other contexts, numerous groups that experience persistent collective victimization advocate for recognition of their strengths, resilience, and perseverance [39]. While such a discourse is more likely to gain the respect of outgroups [4,21], a concern is that presenting the group from a strength-based perspective might undermine support for actions that would challenge the status hierarchy [6], along with diminishing allies’ perceptions that outside intervention is necessary or effective [22]. Some have further suggested that a power advantage could be seen as a moral disadvantage [28,38]. While this might be true, in the present study, the salience of a nation’s strength did not seem to have consequences, favorable or otherwise, for the perceived legitimacy of its actions to protect its identity from existential threats.

The present investigation assessed the perceptions of citizens of a third-party observer nation, without assessing the views of citizens of the nations in conflict, namely Americans or Israelis themselves. It might well be that the political spin that leaders use to account for their conflict actions are more effective in mobilizing ingroup support. For example, when ingroup members encounter a threat, group identification is increased [40]. Perceiving the ingroup as strong and resilient may create the basis for collective pride [41], and group members are more likely to follow and be inspired by a leader who is viewed as triumphant in the face of adversity [4]. Taken to an extreme, ingroup glorification can further serve to minimize ingroup members’ recognition of the emotional suffering of their opponent and to dehumanize them, thereby reducing collective guilt and justifying their own group’s actions [42,43]. Thus, the narrative of President Trump to ‘make America great again’, particularly in the face of threats from an apparently hostile international community, might well be effective in strengthening support among his domestic following, even as it alienates American allies. Indeed, followers might otherwise be tempted into complacency, or might even protest actions that, without such a narrative, they would otherwise perceive as unjust or too costly to the well-being of the ingroup.

Despite Canadians diminished identification with their American neighbour in 2018, the relations between perceptions of the vulnerability of the nations and the justifiability of their conflict actions remained. Assessing these issues over time has important implications for understanding shifts in the views of nations involved in ongoing conflicts. In particular, both the U.S. and Israel were perceived to be less vulnerable and to be better positioned to protect themselves in 2018. This shift might have especially important implications for Israel, as perceived vulnerability was associated with observers’ perceptions that conflict actions were morally justifiable. As the Arab-dominated United Nations continues to single out Israel, and anti-Semitism continues to rise globally [44], knowledge of the historical victimization of Jews is dissipating among younger generations in Europe and North America. For example, in the U.S., 22% of millennials (aged 18–34) said they had never heard of, or were unsure whether they had heard of, the Holocaust [45]. For better or worse, as observers’ understanding of the political history and relationships of groups involved in intractable conflicts shift, so too will allegiances and expectations of behaviour, possibly resulting in political interventions that contribute to historical repetition of victim persecution.

In conclusion, not surprisingly, for a host of reasons, it appears that nations in conflict vary in the perceptions they elicit from citizens of an observer country. To the extent that the safety and security of such nations is viewed to be at risk, their conflict actions are regarded as more morally justified, and on the whole, nations’ power or strength to contend with threats to their national security did not mitigate or enhance observers’ perceptions of the legitimacy of their actions. Such an understanding might entice top dog nations to engage in victim narratives to increase perceptions of their vulnerability. In actuality the present findings suggest that victim narratives may be more critical to observers’ perceptions of less powerful nations in conflict, whereas highlighting shared identity with allies may work more to the benefit of superpower nations.

## Supporting information

S1 File Minimum data for Study 1. These data are in SPSS format.
(SAV)

S2 File Minimum data for Study 2. These data are in SPSS format.
(SAV)

S3 File Minimum data for Study 3. These data are in SPSS format.
(SAV)
